# Activation of Kras^G12D^ in Subset of Alveolar Type II Cells Enhances Cellular Plasticity in Lung Adenocarcinoma

**DOI:** 10.1158/2767-9764.CRC-22-0408

**Published:** 2023-11-24

**Authors:** Priyanka Chaudhary, Xia Xu, Guangfang Wang, Jacob P. Hoj, Rishi R. Rampersad, Marie-Liesse Asselin-Labat, Stephanie Ting, William Kim, Pablo Tamayo, Ann Marie Pendergast, Mark W. Onaitis

**Affiliations:** 1 Moores Cancer Center, University of California San Diego, San Diego, California.; 2 Department of Surgery, University of California San Diego, San Diego, California.; 3 Department of Surgery, Duke Medicine, Durham, North California.; 4 Department of Pharmacology and Cancer Biology, Duke Medicine, Durham, North California.

## Abstract

We have previously identified alveolar type II cell as the cell-of-origin of Kras^G12D^-induced lung adenocarcinoma using cell lineage–specific inducible Cre mouse models. Using gain-of-function and loss-of-function genetic models, we discovered that active Notch signaling and low Sox2 levels dictate the ability of type II cells to proliferate and progress into lung adenocarcinoma upon Kras^G12D^ activation. Here, we examine the phenotype of type II cells after Kras activation and find evidence for proliferation of cells that coexpress type I and type II markers. Three-dimensional organoid culture and transplantation studies determine that these dual-positive cells are highly plastic and tumor initiating *in vivo*. RNA sequencing analysis reveals that these dual-positive cells are enriched in Ras/MAPK, EGFR, and Notch pathways. Furthermore, the proliferation of these cells requires active Notch signaling and is inhibited by genetic/chemical Sox2 upregulation. Our findings could provide new therapeutic strategies to target KRAS-activated lung adenocarcinomas.

**Significance:**

Identification of progenitor like tumor-initiating cells in KRAS-mutant lung adenocarcinoma may allow development of novel targeted therapeutics.

## Introduction

Lung adenocarcinoma is the most common histologic type of non–small cell lung cancer and displays high rates of somatic mutation and genomic rearrangements (1, 2). The main oncogenic driver genes found in lung adenocarcinomas are *KRAS, EGFR, ALK, BRAF, HER2, PIK3CA, ROS1*, and *RET* (3, 4). Among these, *KRAS* mutations are the most common oncogenic aberrations found in almost 25%–30% of lung adenocarcinoma cases, and were the first genetic lesions identified in lung adenocarcinoma more than 30 years ago (5, 6). Despite these facts, only limited treatment options are available for *KRAS*-mutant patients. Development of targeted therapies has only progressed for the G12C mutant, and numerous reports demonstrate that lung adenocarcinoma with *KRAS^G12D^* mutations have poor overall survival (7, 8). Therefore, there is an urgent need to identify novel intervention therapies.

We have previously demonstrated that type II cells in the alveoli of the mouse lung are a cell of origin of Kras-induced lung adenocarcinoma (9). Subsequently, we found that active Notch signaling and downregulation of Sox2 are necessary for Kras-induced tumor formation (10). However, while these changes determine the ability of the cell to transform into lung adenocarcinoma, little is known about the underlying cellular mechanisms.

There has been considerable progress over the past decade in our understanding of lung development (11, 12). Primary lung buds form from the anterior foregut endoderm at E9.5 and branching morphogenesis then occurs between E9.5 and E16.5. This process involves complex cellular and molecular interactions between the epithelial endoderm and surrounding mesoderm. The canalicular and saccular stages follow during which the terminal buds narrow and develop sacs that are the precursors of the alveoli, and the final process of alveolization occurs from P5-30. During the pseudoglandular stage, the cells in the tips of the buds are multipotent progenitor cells that express Id2. Lineage tracing of these cells demonstrates that between E11.5 and E13.5 they give rise to descendants in both proximal airways (Club, ciliated, and neuroendocrine cells), and alveoli (type II and type I) cells. However, Id2+ cells labeled at E17.5 only give rise to alveolar cells (13). In addition, there is some evidence that type I and type II cells arise from bipotent progenitor cells present at E18.5 through a series of intermediate progenitors (14, 15). However, others have argued that the commitment of progenitor cells to either a type II or type I fate occurs earlier and that most distal tip cells at E18.5 are no longer bipotential (16, 17). Recently, some evidence for Kras^G12D^-mediated loss of lineage identity and reprogramming of type II cells into diverse cell states with distinct transcriptional signature was provided by utilizing single-cell RNA sequencing (scRNA-seq) in genetically engineered mouse models (GEMM; refs. 18–20). Each of these studies demonstrates loss of type II cell markers and upregulation of type I cell markers. However, the phenotypic effects of these events on tumor initiation *in vitro* and *in vivo* are unclear.

In the current article, we attempt to understand cellular phenotype of Kras^G12D^ induction using GEMMs. We demonstrate that Kras activation in mature lung type II cells leads to the appearance of cells that coexpress markers of both type II cells (Sftpc) and type I cells (Rage). These dual-positive, type I/II+ cells are tumor-propagating cells *in vitro* and *in vivo.* We provide further evidence that Notch signaling is required for this process while genetic and chemical upregulation of Sox2 profoundly inhibits dedifferentiation and tumor formation. RNA-seq analysis of these dual-positive cells reveals that these cells share pathways with embryonic lung stem cells and express many potential therapeutic targets. Using *in vitro* three-dimensional (3D) organoid cultures derived from Kras-activated type II cells, we demonstrate small molecules that specifically inhibit Kras^G12D^-driven tumor sphere formation. Overall, our findings could provide novel therapeutic strategies to target KRAS-activated lung adenocarcinomas.

## Materials and Methods

### Mice

The *Sftpc-CreER; Rosa26-Tdtomato*, *Sftpc-CreER; LSL-K-Ras^G12D^; Rosa26-Tdtomato,* and *Rosa26-LSL-CAG-Sox2* mouse lines have been described previously (9, 21). *CBF:H2B-Venus* mice (22) were from the Jackson laboratories. *Rosa26-LSL-DNMaml-GFP* mice (23) were kindly provided by Warren Pear (University of Pennsylvania, Philadelphia, PA). All animal experiments were approved by the Institutional Animal Care and Use Committees of both Duke University (Durham, NC) and University of California San Diego (San Diego, CA).

### Tamoxifen/RepSox Administration to Mice

A total of 20 mg/mL tamoxifen (Sigma) dissolved in corn oil was intraperitoneally injected in 6-week-old mice at 75 mg per kg body weight. A total of 0.5 mmol/L RepSox (Sigma) was prepared in PBS and administered intraperitoneally after 1 week of tamoxifen treatment for 3 weeks (two times/week) at 0.25 mg/g body weight.

### Histology and Immunostaining

After indicated timepoints, animals were euthanized and lungs were harvested as described previously (10). Briefly, lungs were perfused with PBS and then fixed with 4% paraformaldehyde overnight. Paraffin sections (7 mm) were made after dehydration and paraffin embedding. Immunofluorescence staining was performed on paraffin-embedded sections using following primary antibodies: rabbit anti-proSftpc (1:500, Millipore, #AB3786), rat anti-Rage (1:200, R&D Systems, #MAB1179), chicken anti-GFP (1:400, Aves Labs, #GFP1020), rabbit anti-Krt5 (1:500, Covance), rabbit anti-CC10 (1:10,000, Barry Stripp), rabbit anti-Sox2 (1:250, Cell Signaling Technology, #3579), rabbit anti-TGFβ (1;200, Protein Tech, #21898-1-AP), mouse anti-TTF1 (1:200, Dako, #8G7G3/1), and rabbit anti-Ki67 (1:500, Cell Signaling Technology, #12202). Alexa Fluor-coupled secondary antibodies (Invitrogen) were used at 1:500 dilution. DAPI (Cell Signaling Technology, 4083) was used to stain nuclei at a concentration of 0.1 mmol/L. Images were captured on a Leica Sp2 laser scanning or Nikon A1R confocal microscope.

### Cell Isolation and Flow Cytometry

*Sftpc-CreER; Rosa-26-TdTomato* and *Sftpc-CreER; LSL-Kras^G12D^; Rosa-26-TdTomato* mice were sacrificed 4 weeks after tamoxifen injection. Lungs were perfused with 10 mL of PBS and lavaged four times with 1 mL of PBS and 0.2 µmol/L Ethylene glycol tetraacetic acid (EGTA). Lung lobes were then minced and incubated with 2 mg/mL collagenase (Worthington Biochemicals) and 1 mg/mL DNase (Worthington Biochemicals) in 0.2 g/L glucose containing DPBS for 1 hour at 37°C. Cells were passed through a 40 µm cell strainer and red blood cells (RBC) lysed using RBC lysing buffer (eBioscience Inc.). Isolated cells were resuspended at 1 × 10^6^ cells in 40 µL of 2% FBS containing DPBS. Blocking was performed with anti-FcR (CD16/CD32; 1:80) and rat-IgG (1:10) at 4°C for 10 minutes. Cells were then incubated with following primary antibodies: CD31-biotinylated (1:50, eBioscience, #13-0311), CD45-biotinylated (1:200, eBioscience, #13-0451), SPC (1:00, Thermo Fisher Scientific, #1bs-10067R), and PE/Cy7 anti-human podoplanin antibody (1:500, BioLegend, #337013). Secondary antibody used was streptavidin-Phycoerythrin/Cy5 (1:500, BioLegend, #405205). DAPI was used for dead cell discrimination. Epithelial cells were selected by depleting CD31^+^ and CD45^+^ population. These epithelial cells were further sorted to three different populations based on Podoplanin (PDPN) and TdTomato staining: type I (PDPN^+^) and type II (TdTm+) and double-positive (PDPN+ TdTm+). FACS was performed on FACSAria II and analysis was done with FlowJo.

### Quantitative Real-time PCR

RNA was extracted from sorted double-positive population (PDPN+ TdTm+) and total epithelial Cells using RNeasy mini kit (Qiagen, #74104) as per the manufacturer's instruction. To eliminate residual DNA from the sample, DNase I (Qiagen, #79254) treatment (15 minutes at room temperature) was done over the column during RNA preparation. The cDNA was prepared using SuperSript IV reverse transcriptase first-strand synthesis system (Invitrogen, #18091050). A total of 10 µL reaction mixtures containing cDNA template, SYBR Green PCR mastermix (Applied Biosystems, #4344463) and 10 pmol of each of GAPDH (housekeeping gene) or respective gene-specific oligonucleotide primer pairs (Supplementary Table S1) were run on a QuantStudio 7 Flex Real-Time PCR System (Applied Biosystems) using the following program: initial denaturation at 95°C for 5 minutes and 40 cycles of 95°C for 30 seconds, 55°C for 30 seconds, and 72°C for 30 seconds followed by the melt curve analysis. The fold change in the target gene relative to housekeeping gene (GAPDH) was calculated as: Fold Change = 2^−d(dCT)^, where dCT = CT _(target)_ − CT_(GAPDH)_ and d(dCT) = dCT _(Treated)_ − CT _(control)_.

### RNA-seq

RNA was extracted from sorted type I, type II, and double-positive population using RNeasy mini kit as described previously. Library preparation and RNA-seq was carried out at Duke University Genome Sequencing and Analysis Core. Briefly, quality of RNA was assessed using TapeStation systems (Agilent technologies). RNA-seq libraries were prepared from total RNA using TruSeq Stranded kit (Illumina) and separated as 75 bp paired-end reads using HiSeq4000. Reads were then aligned to mouse reference genome.

### Downstream Data Analysis

To perform an unbiased clustering of the data, we performed non-negative matrix factorization (NMF; refs. 24, 25) across the RNA-seq dataset containing nine samples. To evaluate the enrichment of different cell types during mouse lung development, we generated cell type–specific signatures (gene sets) for E11.5, E17.5 lung progenitor, and differentiated epithelium derived from Gene Expression Omnibus (GEO) datasets GSE75860, GSE111303, and GSE111300 by selecting the top 100 most differentially expressed genes for each cell type. Enrichment scores for the corresponding samples were calculated using single-sample GSEA (ssGSEA; ref. 26). Briefly, gene expression values in each individual sample were rank normalized by their absolute expression, followed by calculation of an enrichment score calculated by evaluating the differences in the empirical cumulative distribution functions of the genes in the gene set relative to the remaining genes. A positive enrichment score denotes significant overlap of the gene set with groups of genes at the top of the ranked list, while a negative enrichment score denotes a significant overlap of the signature gene set with groups of genes at the bottom of the ranked list. These resulting ssGSEA scores were used to assess enrichment of cell-type signatures in each group. To quantify the degree of association, an information-theoretic measure, the information coefficient (IC), was calculated and an empirical permutation test for statistical significance calculations carried out (27). We also performed a systematic evaluation of more general gene sets that associate with the PdTm group by performing ssGSEA using gene sets from the Molecular Signatures Database (MSigDB, subcollections: C2, C3, C6). Among the top matching gene sets, we focused on multiple independent gene sets that represent the same pathway or biological process. An Onco-GPS map was generated by performing NMF decomposition of 1,000 genes induced or repressed by the activation of KRAS G12V in immortalized lung epithelial cells (28) across samples from the Broad-Novartis Cell Line Encyclopedia Reference Dataset (29).

### Organoid Culture and Drug Treatments

Sorted type I, type II, and double-positive cells were resuspended in 45 µL MTEC/Plus media (30), mixed with equal volume of growth factor reduced Matrigel (BD Biosciences, #356231) and plated in a 24-well 0.4 µm transwell insert (Falcon, #353095) with seeding density of 5,000 alveolar cells per insert. A total of 10^5^ PDGFR-GFP^hi^ feeder cells were also added to each insert. A total of 500 µL MTEC/Plus was placed in the lower chamber and medium was changed every other day. A total of 10 µmol/L ROCK inhibitor (Sigma, #Y0503) was added to the medium for the first 2 days of culture. For drug treatment experiments, RepSox (Sigma), dibenzazepine (DBZ; Calbiochem), and SB-431542 (Sigma; TGFβ inhibitor) were used at a concentration of 2 mmol/L. DBZ and SB were prepared by suspending in DMSO while RepSox was dissolved in PBS. In all *in vitro* experiments, drugs were added on day 3 after initial plating cells in cell culture dishes. Fresh media with drugs/inhibitors was added every other day for 21 days.

### Transplantation Assay in Mice

Type II+ and double-positive (type I/II+) cells were sorted using flowcytometry. A total of 25,000 type II+ or double-positive cells were transplanted into one flank of either *Rag1^−^^/^^−^* or NOD/SCID mice subcutaneously. Sorted cells were resuspended in 100 µL mixture of 1:1 ratio of PBS to Matrigel and administered subcutaneously into the flank of immunocompromised mice. Palpable tumors were observed in about 2 months after the transplantation. Tumors were harvested 3 months after transplantation and histology core was used for sections preparation and hematoxylin and eosin (H&E) staining.

### Cell and Tumor Quantification

ImageJ software (NIH, Bethesda, MD) was used for quantitative analysis of colony number and colony area for *in vitro* culture experiments using an algorithm to identify all roughly circular cell masses greater than 2,000 (mm)^2^ in area. Tumor counts were also obtained with ImageJ software using H&E-stained section. The percentage of tumor per section was calculated as the sum of the area of all tumors divided by the area of the entire lung on the section.

### Statistical Analysis

Data represent mean ± SEM. Statistical analysis of the experimental data was performed using Student *t* test of GraphPad Prism software. The level of significance is shown in the figures with asterisks and defined as *, *P* ≤ 0.05; **, *P* ≤ 0.01; and ***, *P* ≤ 0.001.

### Data Availability

The RNA-seq data generated in this study are publicly available in GEO at GSE230664. Rest of the data generated in this study are available upon request from the corresponding author.

## Results

### Activation of Kras^G12D^ in Type II Cells Leads to a Proliferation of a Population of Cells Expressing Both Type II and Type I Markers

**FIGURE 1:**
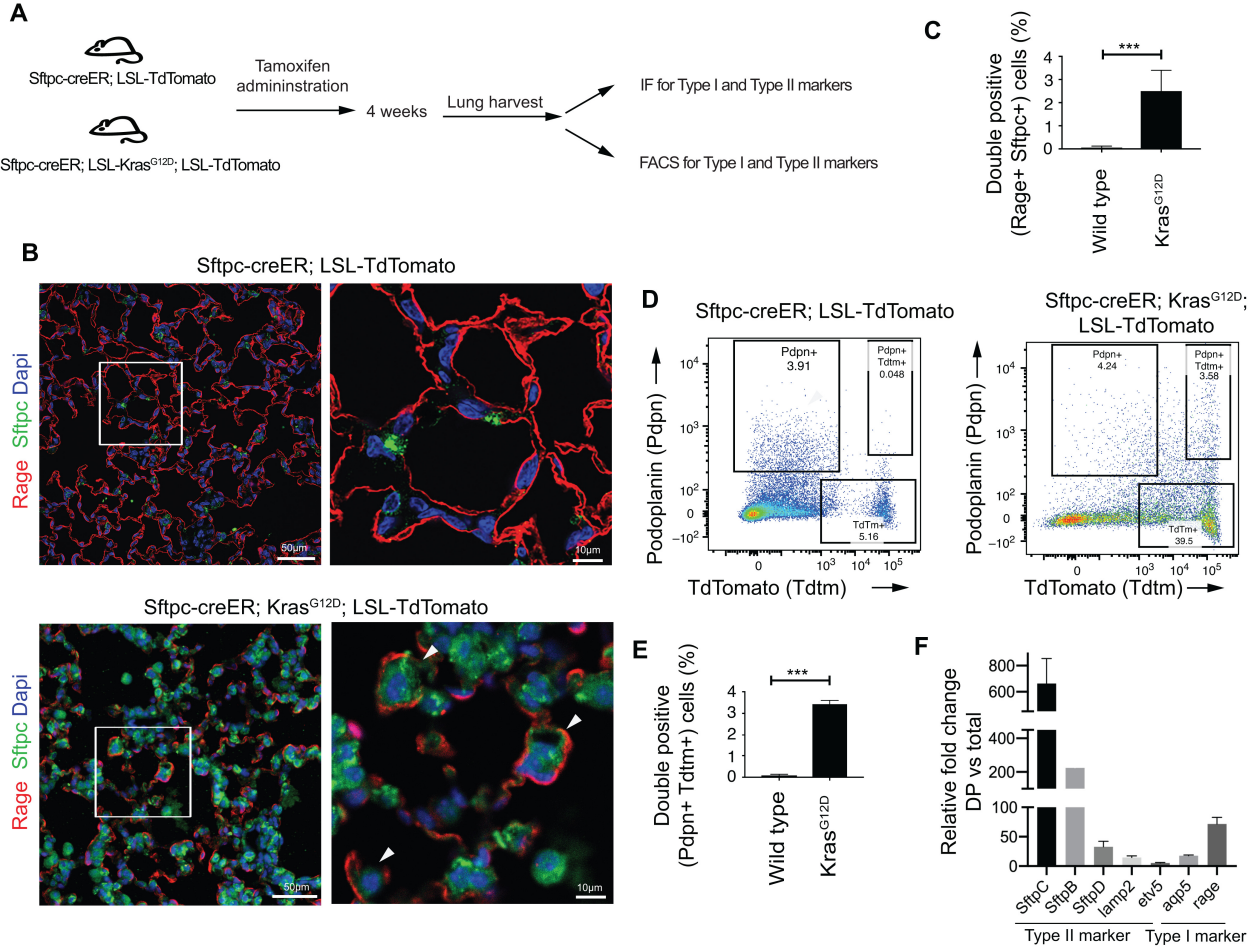
Activation of Kras^G12D^ transforms type II cells to dual-positive type I and type II expressing cells. **A,** Experimental outline to compare type I, type II, and double-positive (type I/II+) population between wild-type and Kras^G12D^-mutant mice. **B,** Immunofluorescent images showing sections of left lobes of *Sftpc-CreER; Rosa26-TdTomato* (top) and *Sftpc-CreER; LSL-Kras^G12D^; Rosa26-TdTomato* (bottom) mice, 4 weeks after single dose of tamoxifen injection stained with DAPI (blue), Sftpc (green) and Rage (red). Right panel shows higher magnification of boxed area in left panels. Note that many tumor cells are Sftpc+Rage+ (marked with white arrows) in Kras-mutant mice. **C,** Quantitative analysis of B. **D,** FACS analysis for comparing type I, type II, and double-positive (type I/II+) population between wild-type and Kras^G12D^ mice. **E,** Quantitative analysis of D. **F,** qPCR analysis for comparing the gene expression of some of the type I marker and type II marker genes between double-positive cells and total epithelial cells from *Sftpc-CreER; Kras^G12D^; TdTomato* mice. Results shown in B and D are representative of three independent experiments. C, E, and F represent data from three independent experiments. Error bars represent the mean ± SEM values and significance is defined as *, *P* ≤ 0.05; **, *P* ≤ 0.01; and ***, *P* ≤ 0.001.

To examine the early cellular consequences of Kras activation in type II cells, we generated Kras-activated lung tumors by giving a single dose of tamoxifen to *Sftpc-CreER; LSL-Kras^G12D^; Rosa-26-TdTomato* (experimental) or *Sftpc-CreER; Rosa-26-TdTomato* (control) mice. At 4 weeks, the lungs were fixed, sectioned, and immunostained for type I and type II markers (Fig. 1A). Multiple type I and type II markers have been identified and validated (Supplementary Table S2; refs. 14, 15). We used anti-Rage and anti-Pro-Sftpc antibodies to label type I and type II cells, respectively. Our results show that lung sections from the control *Sftpc-CreER; Rosa-26-TdTomato* mice mostly contain either type I cells (Rage+) or type II cells (Sftpc+), and only 0.05%± 0.07%, Sftpc+Rage+ cells of total epithelial cells (Fig. 1B, top and Fig. 1C). By contrast, *Sftpc-CreER; LSL-Kras^G12D^; Rosa-26-TdTomato* lungs contain significantly more dual-positive (Sftpc+Rage+) cells (2.6% ± 0.9% of total epithelial cells, *P* < 0.05; Fig. 1B, bottom and Fig. 1C).

Next, we performed flow cytometry to quantify these double-positive cells along with single-positive, type I and type II cells. We administered tamoxifen to *Sftpc-CreER; Rosa-26-TdTomato* and *Sftpc-CreER; LSL-Kras^G12D^; Rosa-26-TdTomato* mice and harvested lungs after 4 weeks. Lung tissue was digested and stained with CD31, CD45, and PDPN (type I marker) antibodies. Quantification of the total number of single type I+ [Podoplanin (PdPn+)], type II+ [TdTomato (Tdtm)], and double-positive cells was performed in the CD31^−^ CD45^−^ population. Our results demonstrate that control mice express remarkably few PdPn+Tdtm+ (double-positive) cells (0.048%) compared with Kras-activated mice (Fig. 1D and E).

We next tracked development and proliferation of these double-positive cells from Kras-activated mice after tamoxifen administration (Supplementary Fig. S1). Our flow cytometry data corroborated immunofluorescence results demonstrating that Kras activation leads to enrichment of double-positive (type I/II+) population in the lung alveolar region.

To further examine these double-positive cells, we sorted these PdPn+Tdtm+ (double-positive) cells using FACS and assessed the expression of type I and type II cell markers using qPCR. Our results show that both type I (Rage, Aqp5) and type II markers (Sftpc B, Sftpc C, Sftpc D, lamp2, and etv5) are enriched in double-positive cells compared with total unsorted cells (Fig. 1F), corroborating that the double-positive cells express both type I and type II markers.

### Type I/II+ Dual-positive Cells in Sftpc-CreER; Kras^G12D^ Lungs After Kras Activation are Tumor-propagating and Forms Tumors Upon Transplantation

**FIGURE 2:**
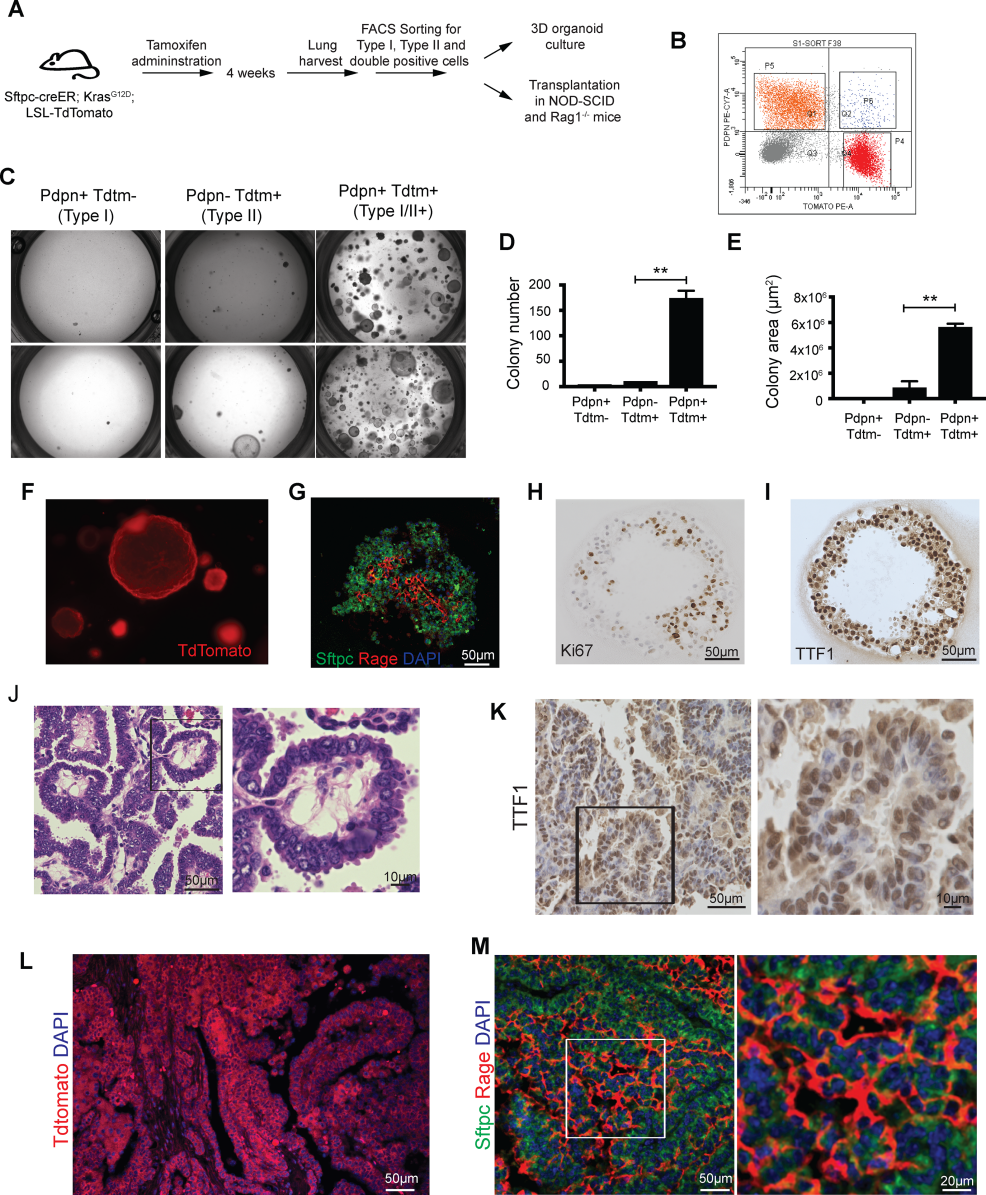
Double-positive cells in *Sftpc-CreER; LSL-Kras^G12D^*-induced lung adenocarcinoma are tumor-propagating cells and forms tumors upon transplantation. **A,** Experimental outline for growing cells in 3D organoid culture. **B,** FACS scatter plot showing single (type I or type II) and double-positive population sorted from *Sftpc-CreER; LSL-Kras^G12D^; TdTomato* mice. Type I (TdTm−Pdpn+), type II (TdTm+ Pdpn−), and double-positive (type I/II+; TdTm+Pdpn+) fractions were sorted. **C,** Brightfield images of 3D organoid cultures from different indicated sorted populations on day 14. Colony number (**D**) and colony size (**E**) was quantified using ImageJ software. **F,** 3D organoid culture from double-positive cells on day 14 in red channel. **G,** Immunofluorescent image on day 14 of organoid culture showing double-positive cells differentiate into type I and type II cells. Ki67 (**H**) and TTF1 (**I**) staining of 3D organoid culture. **J,** Representative H&E staining of tumors sections which were obtained after subcutaneous transplantation of double-positive (type I/II+) cells. Right panel shows higher magnification of boxed area in left panels. **K,** Representative IHC images of tumor sections for TTF1. Right panel shows higher magnification of boxed area in left panels. **L,** Representative immunofluorescent image for in red channel showing Tdtomato expression in tumor obtained after transplantation. **M,** Representative IF images for Sftpc and Rage staining. Right panel shows higher magnification of boxed area in left panels. Results shown in B, F, G, H, I, J, K, L, and M are representative of three independent experiments. D and E represent data from three independent experiments. Error bars represent the mean ± SEM values and significance is defined as *, *P* ≤ 0.05; **, *P* ≤ 0.01; and ***, *P* ≤ 0.001.

We next tested the ability of single- and double-positive sorted populations to proliferate in culture. Figure 2A demonstrates the experimental outline. Utilizing a previously established clonal 3D coculture system in which type II cells are combined with Pdgfra+ mesenchymal cells (31), we assayed the colony-forming efficiency of Kras-mutant single (Pdpn+Tdtm− and Pdpn−Tdtm+) and dual-positive (Pdpn+Tdtm+) cells. Type I (Pdpn+Tdtm−), type II (Pdpn−Tdtm+), and dual-positive (Pdpn+Tdtm+) cells from *Sftpc-CreER; LSL-Kras^G12D^; Rosa-26-TdTomato* animals after 4 weeks of tamoxifen administration were sorted using flow cytometry (Fig. 2B). Sorted cells were seeded at a density of 5,000 cells in 90 µL of culture medium with 50% Matrigel in 24-well Transwell inserts together with 1 × 10^5^ PDGFRA-GFP stromal cells freshly sorted from lungs of *Pdgfra-H2B; GFP* transgenic mice. After 14 days, spheres of lineage-labeled cells are present in various sizes. As Fig. 2C demonstrates, double-positive (Pdpn+Tdtm+) cells form colonies much more efficiently than Kras-mutant type II cells that do not express type I markers. Furthermore, colony size is significantly larger in the double-positive cell cultures (Fig. 2D and E). Fluorescent microscopic images of these 3D organoid cultures from double-positive cells in red channel show that these organoids express TdTomato, the lineage label for type II cells in our genetically modified mice (Fig. 2F).

Previously, it was reported that 3D organoid cultures from type II cells can differentiate into both type I and type II cells (31). To investigate whether double-positive cells result in multiple cell types, we stained 14-day spheres with Rage and Sftpc antibodies. Confocal microscopy images show that the 3D spheres from double-positive cells contain single-positive (Rage) and Sftpc cells (Fig. 2G). In addition, IHC analysis shows positive staining for Ki67 demonstrating active proliferation in the cultures (Fig. 2H). Moreover, the organoid cultures express type II cell marker, TTF1 (Fig. 2I). Overall, our results demonstrate that the “dual-positive” (type I/II+) cells have high colony formation efficiency in 3D cultures, are actively proliferating, and can differentiate into type I and type II cells.

To ascertain whether the *in vitro* growth and proliferative advantages of the dual-positive cells are also seen *in vivo*, we transplanted type II+, PdPn- and double-positive (type I/II+) cells into immunodeficient Rag1^−/−^ and NOD/SCID mice in the flank subcutaneously. As Table 1 demonstrates, allografts with dual-positive cells develop significantly higher number of tumors in both Rag1^−/−^ (7 tumors out of 10 transplants) and NOD/SCID (8 tumors out of 9 transplants) mice compared with Kras-mutant type II cells that do not express type I markers (2 tumors out of 10 transplants in Rag1^−/−^ and 0 tumor out of 8 transplants in NOD/SCID) suggesting that the dual-positive (type I/II+) cells preferentially propagate tumors *in vivo*. The tumor images from both *Rag1*^−/−^ and NOD/SCID mice are shown in Supplementary Fig. S2. The H&E staining shows that tumors are papillary tumors, similar to *in situ* tumors that develop in *Sftpc-CreER; LSL-Kras^G12D^* mice (Fig. 2J; Supplementary Fig. S3). These tumors also express TTF1, the adenocarcinoma marker (Fig. 2K) and Tdtomato, the lineage label for type II cells (Fig. 2L). We also performed immunofluorescence staining for type I and type II cells on these tumor sections, and they contain both type I (Rage) and type II (Sftpc) cells (Fig. 2M; Supplementary Fig. S4), suggesting that these dual-positive (type I/II+) cells differentiate into type I and type II cells upon transplantation. Taken together, our results demonstrate that the double-positive (type I/II+) cells are efficient tumor-initiating cells *in vivo.*

**TABLE 1:** Number of subcutaneous tumors resulting after 8 weeks of engraftment on the flank of immunodeficient mice

	Tumors found (Number of tumors/Number of transplants carried out)	
Mouse background	Kras^G12D^ type II cells	Kras^G12D^ dual-positive (type I/II^+^) cells
Rag1^−/−^	2/10	7/10
NOD SCID	0/8	8/9

### The Kras^G12D^-induced Upregulation of Type I Cell Markers in Double-positive Cells Requires Notch Signaling

**FIGURE 3:**
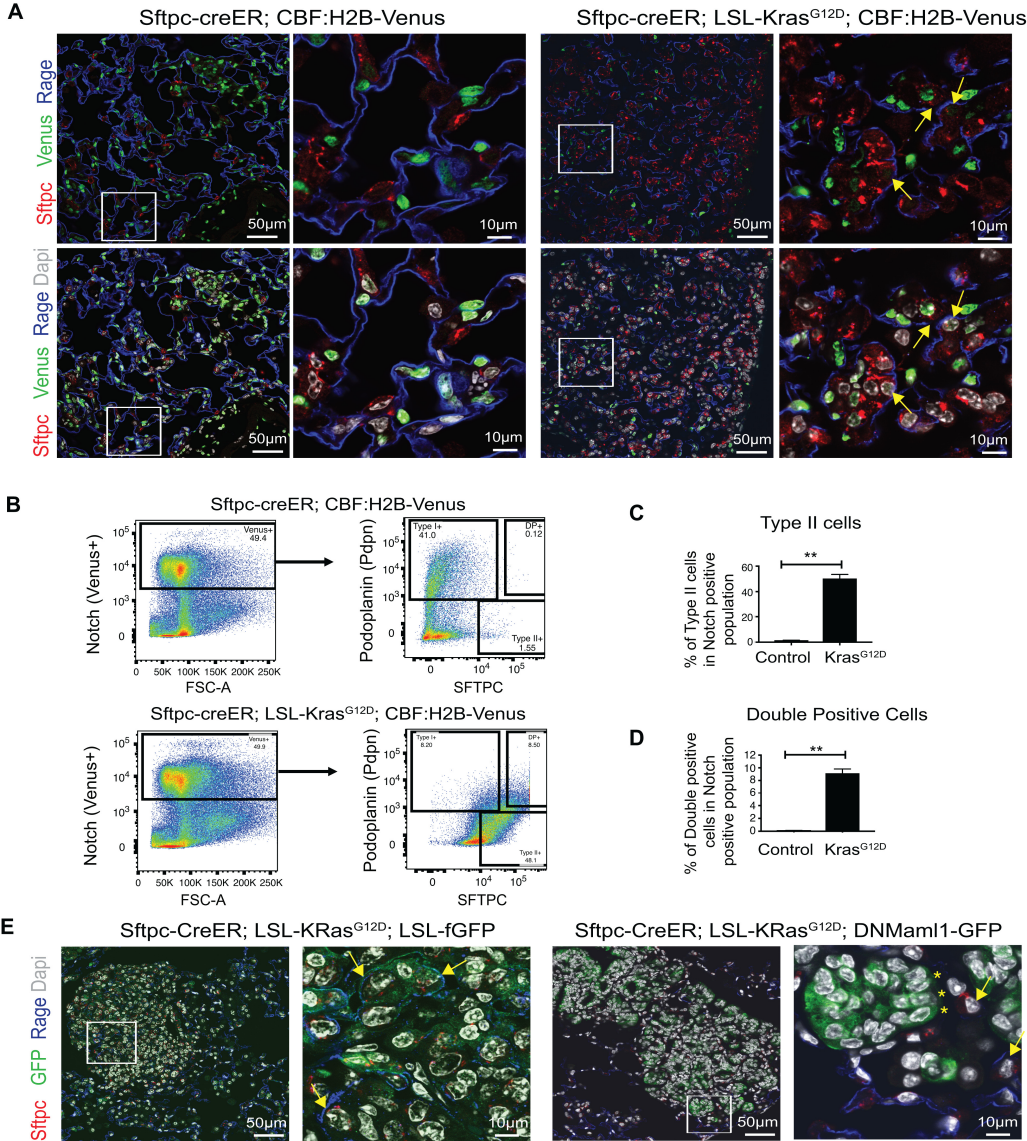
The Kras^G12D^-induced upregulation of type I cell markers in double-positive cells requires Notch signaling. **A,** Representative IF image of lung sections of indicated genotypes stained with GFP (Venus, Notch signaling reporter), Sftpc (red) and Rage (blue) staining in mouse*.* Note that Sftpc+Rage+ tumor cells express Venus (Notch reporter; marked with yellow arrows). **B,** Representative FACS density plots showing Notch+ population (left) in wild-type (*Sftpc-CreER; CBF-H2B-Venus*) versus mutant Kras (*Sftpc-CreER; CBF-H2B-Venus; LSL-Kras^G12D^*) mice. FACS plots on right side display type I and type II population from gated Notch+ population in wild-type and mutant Kras mice. DP, double positive. **C,** Quantification of type II cells in Notch+ population between wild-type and Kras-mutant mice. **D,** Quantification of double-positive (type I/II+) cells in Notch+ population between wild-type and Kras-mutant mice. **E,** Representative IF image of lung sections of indicated genotypes stained with GFP (green), Sftpc (red), and Rage (blue). Note that Sftpc+Rage+ tumor cells (marked with yellow arrows) are visible in *Sftpc-CreER; Kras^G12D^; Rosa-fGFP* mice but absent in GFP+ cells in *Sftpc-CreER; LSL-Kras^G12D^; DNMaml1* mice. Results shown in A, B, and E are representative of three independent experiments. C and D represent data from three independent experiments. Error bars represent the mean ± SEM values and significance is defined as *, *P* ≤ 0.05; **, *P* ≤ 0.01; and ***, *P* ≤ 0.001.

We have previously demonstrated the necessity for Notch activation downstream of Kras for adenocarcinoma formation in distal lung cells (10). To test whether Notch signaling is activated in double-positive (TdTomato+PdPn+) cells after Kras activation, we crossed *Sftpc-CreER; LSL-Kras^G12D^* mice to CBF-H2B-Venus (Notch reporter mice; ref. 22). Lungs were harvested 4 weeks after tamoxifen administration, and staining was performed for type I and type II markers. As shown in Fig. 3A, Sftpc+Rage+ tumor cells in *Sftpc-CreER; LSL-Kras^G12D^; CBF-H2B-Venus* express Venus (Notch reporter; yellow arrows; Fig. 3A) suggesting that Notch signaling is activated in double-positive cells.

We next performed quantitative FACS analysis using *Sftpc-CreER; CBF-H2B-Venus* (wild-type) and *Sftpc-CreER; LSL-Kras^G12D^; CBF-H2B-Venus* (mutant) mice. We first compared the overall Notch activation between wild-type and mutant mice by examining the total Venus+ cells. Our results indicate that there is no change in Notch expression between wild-type and Kras-activated mice. However, Notch signaling is significantly upregulated in type II and double-positive cells in Kras-mutant mice compared with wild-type mice (Fig. 3B, right). The FACS analysis reveals that only 1.55% ± 0.005% type II cells in wild-type mice have active Notch compared with 50.35±2.25% type II cells in Kras-mutant mice (Fig. 3C). In addition, 9.16% ± 0.66% double-positive cells (type I/II+) express active Notch in mutant Kras compared with only 0.11% ± 0.01% double-positive cells in wild-type mice (Fig. 3D). These results suggest that Kras^G12D^ activation causes Notch induction in type II and double-positive (type I/II+) cells.

Finally, we inhibited Notch signaling by crossing in a DNMaml1 (dominant negative mastermind-like) allele. We crossed *Sftpc-CreER; LSL-Kras^G12D^* and DNMaml1-GFP mice and generated *Sftpc-CreER; LSL-Kras^G12D^; DNMaml1-GFP* mice. Four weeks after tamoxifen administration, lungs were harvested and stained for Sftpc, GFP, Rage, and DAPI. As Fig. 3E demonstrates, DNMamL1-expressing cells (green, marked by yellow asterisk) do not express type II and type I markers indicating that Notch signaling is necessary for development of these dual-positive cells.

### Genetically Overexpressing Sox2 and Chemically Replacing Sox2 Inhibit Kras-induced Dual-positive Cell Development and Tumor Formation in Alveolar Type II Cells

**FIGURE 4:**
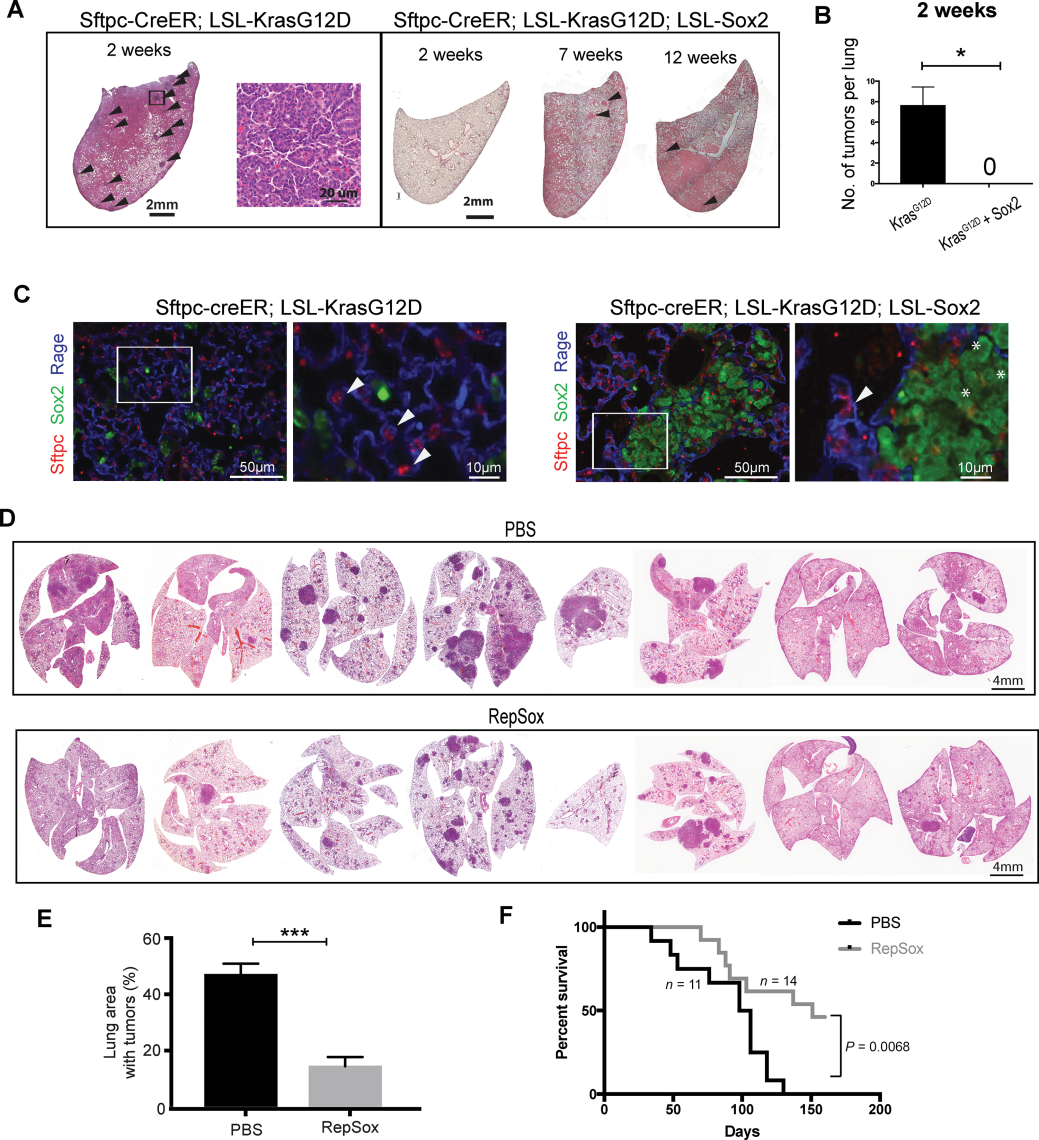
Genetically overexpressing Sox2 and chemically replacing Sox2 inhibit Kras-induced dual-positive cell development and tumor formation in alveolar type II cells. **A,** Sections of *Sftpc-CreER; LSL-Kras^G12D^* and *Sftpc-CreER; LSL-Kras^G12D^; LSL-*Sox2 mouse lungs at 2, 7, and 12 weeks after tamoxifen injection stained with H&E staining. Tumor area is marked with black arrows. *Sftpc-CreER; LSL-Kras^G12D^*—right panel shows higher magnification of boxed area in left panels. **B,** Quantification of tumors observed after 2 weeks after tamoxifen treatment in *Sftpc-CreER; LSL-Kras^G12D^* and *Sftpc-CreER; LSL-Kras^G12D^; LSL-*Sox2 mice. Bar graph represents data from 5 mice from each genotype. **C,** Representative IF images showing Sox2 (green) and Sftpc (red) staining of mouse lung sections in *Sftpc-CreER; LSL-Kras^G12D^* and *Sftpc-CreER; LSL-Kras^G12D^; LSL-Sox2* mice. Note the Sox2+ hyperplastic areas (marked with white asterisks) do not express double-positive markers (Sftpc+/Rage+; right). **D,** Representative H&E images showing the effect of RepSox treatment on the lung of *Sftpc-CreER; LSL-Kras^G12D^; fGFP* mice. RepSox or PBS was administered intraperitoneally for 3 weeks (two times/week) 1 week after tamoxifen treatment. Mouse lungs were harvested after 2 weeks of RepSox treatment. **E,** Quantitative analysis of D using ImageJ software (*n* = 8 for each group). **F,** Kaplan–Meier curve showing survival for RepSox versus PBS treatment in *Sftpc-CreER; LSL-Kras^G12D^; fGFP* mice [(PBS, *n* = 11) and (RepSox, *n* = 14)]. A and B represents data from 5 different mice. Results shown in C are representative of three independent experiments. Error bars represent the mean ± SEM values and significance is defined as *, *P* ≤ 0.05; **, *P* ≤ 0.01; and ***, *P* ≤ 0.001.

We have previously demonstrated that the proximal respiratory epithelial lineage marker Sox2 inhibits Kras-induced lung adenocarcinoma formation potentially due to its inhibitory effect on Notch signaling (10). To ascertain whether Sox2 suppresses development of dual-positive cells when Kras^G12D^ is activated in type II cells, we generated a *Sftpc-CreER; LSL-Kras^G12D^; LSL-Sox2* mouse line and administered tamoxifen at 6 weeks after birth. As shown in Fig. 4A, Sox2-overexpressing mice have markedly smaller tumors than *Sftpc-CreER; LSL-Kras^G12D^* mice at a similar age. *Sftpc-CreER; LSL-KrasG12D* mice develop papillary tumors (Fig. 4A, left, higher magnification image) after 2 weeks of single dose of tamoxifen administration while the lungs of *Sftpc-CreER; LSL-Kras^G12D^; LSL-Sox2* mice appear normal after 2 weeks (Fig. 4A and B). *Sftpc-CreER; LSL-Kras^G12D^; LSL-Sox2* develop hyperplasia and very small tumors (marked with black arrows) between 7 and 12 weeks (Fig. 4A, right). IHC analysis of alveolar hyperplastic regions in *Sftpc-CreER; Kras^G12D^; LSL-Sox2* mice demonstrates strong positive staining for Sox2 (Fig. 4C, right, Sox2+ cells are marked with white asterisks). Furthermore, we found that Sox2 overexpression causes overall reduction in total number of double-positive (Sftpc+Rage+) cells (Fig. 4C, right). Also, Sox2-positive cells express the squamous markers CC10 and Krt5 (Supplementary Fig. S5).

Next, We tested whether the small molecule RepSox that is able to replace Sox2 in cellular reprogramming (32) is able to inhibit Kras-induced tumor formation. We treated *Sftpc-CreER; Kras^G12D^; fGFP* mice with Repsox for 3 weeks 1 week after tamoxifen administration. After 2 weeks of treatment, lungs were harvested and paraffin sections were prepared. IHC analysis demonstrates that RepSox treatment increases Sox2 levels in Kras^G12D^-mutant lungs without any significant change in TGFβ levels (Supplementary Fig. S6). Significantly, our results show that RepSox causes substantial reduction in lung tumor development in *Sftpc-CreER; LSL-Kras^G12D^* mice (Fig. 4D and E). In addition, RepSox administration improved survival of these mice (Fig. 4F). Taken together, our data suggest that Sox2 can act as a tumor suppressor for adenocarcinoma formation in this model at least partly by preventing emergence of dual-positive cells.

### Notch Inhibition or Sox2 Upregulation Suppress Tumor Sphere Formation in 3D Culture

**FIGURE 5:**
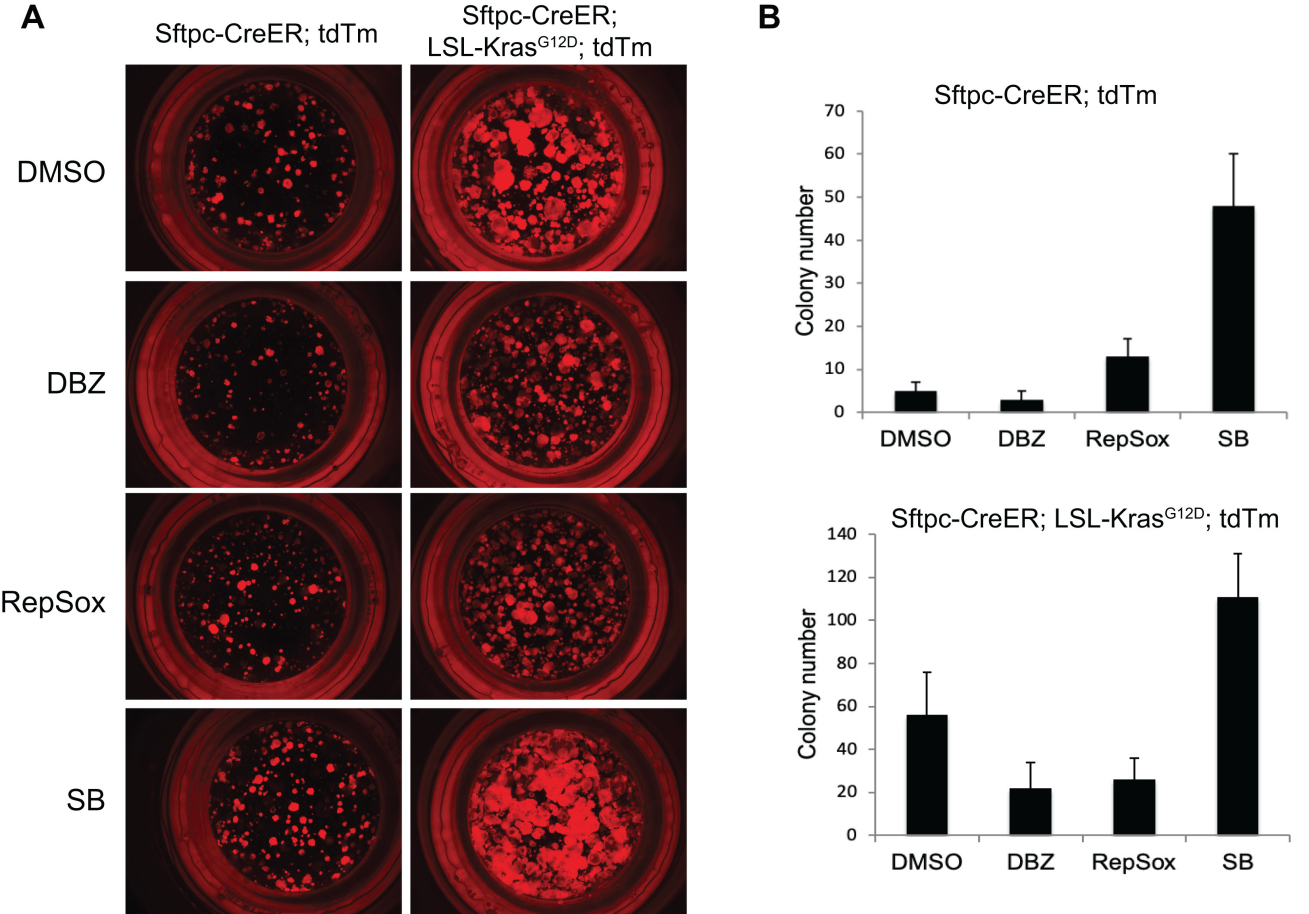
Notch inhibition or Sox2 upregulation suppresses tumor sphere formation in 3D culture. **A,** Representative fluorescent microscopy images of cultures (*n* = 3) showing spheres formed in Matrigel at day 14 from lineage-labeled TdTomato+ type II cells obtained by FACS sorting from *Sftpc-CreER; Rosa-Tdtm* and *Sftpc-CreER; LSL-Kras^G12D^; Rosa-TdTomato* mice*.* Sorting was carried out after 4 weeks of one dose of tamoxifen. **B,** Quantitative image analysis of A showing colony forming efficiency of lineage-labeled cells calculated by ImageJ software. Error bars represent the mean ± SEM values, and significance is defined as *, *P* ≤ 0.05; **, *P* ≤ 0.01; and ***, *P* ≤ 0.001.

Having established the antagonistic role of Notch and Sox2 signaling on Kras-mediated development of the tumor-initiating type I/type II cells marker population, we investigated their effect on *in vitro* colony formation. To do so, we examined the colony formation efficiency of TdTomato+ from lungs of *Sftpc-CreER; Rosa-26-TdTomato* and *Sftpc-CreER; LSL-Kras^G12D^; Rosa-26-TdTomato* in the presence of Notch inhibitor (DBZ) and Sox2 mimetic, RepSox while DMSO was used as control. As expected, we found numerous and larger colonies with Kras activation compared with wild type (*Sftpc-CreER; Rosa-26-TdTomato*) in the DMSO control (Fig. 5A, top). We also observed fewer and smaller colonies with DBZ as well as RepSox treatment in both the genotypes (with and without Kras^G12D^ mutation; Fig. 5A and B), suggesting that Notch inhibition and Sox2 overexpression suppress proliferation of type II cells *in vitro*. We also used SB-431542, a specific inhibitor of TGFβ to analyze the effect of this pathway in these cultures. SB-431542 treatment markedly increases colony number and size. Overall, our results suggest that Notch inhibition and Sox2 overexpression inhibit colony growth *in vitro* in similar fashion to their *in vivo* effects.

### Type I/II+ Dual-positive Cells Share Pathways with Developing Lung Progenitors and Contain Potential Therapeutic Targets

**FIGURE 6:**
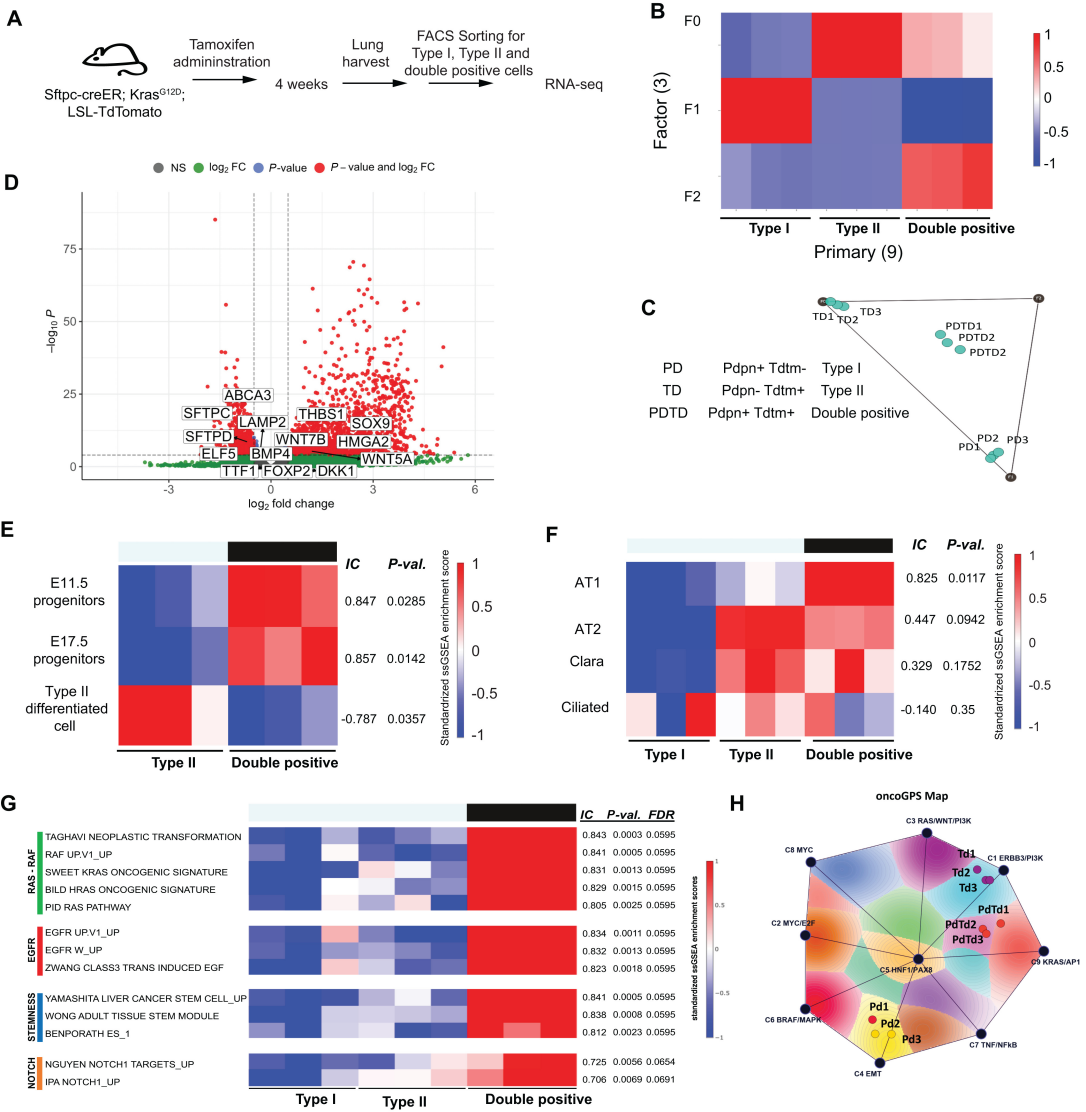
Type I/II+ dual-positive cells share pathways with developing lung progenitors and contain potential therapeutic targets. **A,** Experimental outline for RNA-seq performed with sorted type I, type II, and double-positive cells. **B,** Heat map depicting the results of the NMF analysis of the RNA-seq dataset. The heat map shows the NMF clustering of samples according to cell types, with double-positive cells sharing NMF F0 factor with type II cells (red: enriched, blue: depleted). **C,** 2D Onco-GPS map showing double-positive cells lie between type I and type II cells in NMF-derived transcriptional space. **D,** Volcano plot showing differential gene expression between double-positive and type II cells. Type II marker and embryonic marker genes are highlighted in the plot. **E,** Heat map depicting ssGSEA results comparing current RNA-seq data with previously published datasets containing mouse embryonic and differentiated epithelial cells. Double-positive cells contain gene sets which are significantly enriched in both 11.5 and 17.5 lung progenitors. The scores on the right side are IC measures of association and permutation-test *P* values (see Materials and Methods). **F,** Heat map depicting ssGSEA results comparing current RNA-seq data with scRNA-seq data from mouse embryonic epithelial cells (15). Double-positive cells are significantly enriched in gene sets from AT1, AT2, Club as well as ciliated cells. **G,** Heat map depicting ssGSEA results using gene sets from the MSigDB shows enrichment of multiple gene sets that represent Ras/MAPK, EGFR, and Notch pathways in double-positive cells. **H,** RAS Onco-GPS analysis showing the projection of individual samples represented as color-coded circles. Each color contour represents different cell states associated with RAS activation. The black circles represent the nine RAS nodes representing major oncogenic pathways. Double-positive cells (PdTd) appear to be associated with the RAS/AP1 cell state/pathway.

To further characterize these dual-positive cells at transcriptional level, we performed RNA-seq for single-positive type I cells (Pdpn+, Tdtm−) and type II cells (Pdpn−, Tdtm+); and double-positive cells (Pdpn+, Tdtm+) from *Sftpc-CreER; LSL-Kras^G12D^, LSL-Rosa26-Tdtm* mice after 4 weeks of tamoxifen administration (Fig. 6A). After processing and normalization of RNA-seq data, we first performed NMF analysis to assess how samples will cluster with each other. We observed that each sample clusters with others according to their cell type based on three distinct NMF factors (F0, F1, F2, see Materials and Methods; Fig. 6B). We also find that when sample NMF profiles are further projected onto two-dimensional space for visualization purposes, double-positive cells lie between type I and type II cells, suggesting that double-positive cells share properties of both type I and type II cells (Fig. 6C). Next, we compared the gene expression profile of double-positive cells and type II cells to particularly examine type II and embryonic markers in these populations. Our results demonstrate that many of the embryonic genes (Sox9, HMG2A, THBS1, WNT7B, WNT5A) are enriched while type II markers (SFTPC, SFTPD, ABCA3) are downregulated in double-positive cells (Pdpn+Tdtm+) compared with type II (Tdtm+) cells (Fig. 6D). To further examine similarity to embryonic cell types, we performed ssGSEA (26) using gene sets generated from a previously published dataset (see Materials and Methods) containing Id2+ mouse embryonic (E11.5 and E17.5) and differentiated epithelial cells (13, 33). Our results show that double-positive tumor cells are transcriptionally more similar to embryonic progenitors than the differentiated cell types (Fig. 6E). To further corroborate these findings, we compared our RNA-seq data with previously published scRNA-seq data of 80 embryonic mouse lung epithelial cells (15) and observed that the dual-positive samples were enriched in gene sets of all kinds of embryonic epithelial cells [type I (AT1), type II (AT2), Club as well as ciliated cells] (Fig. 6F).

Finally, we performed a more comprehensive ssGSEA using gene sets from the MSigDB to identify signaling pathways that are enriched in double-positive progenitors relative to other cell types. We found that several gene sets representing Ras/MAPK, EGFR, and Notch pathway were predominantly enriched in the double-positive cells (Fig. 6G). Prior work has demonstrated that RAS-mutant cell states can be categorized into distinct transcriptional states, and that this can be visualized using a novel two-dimensional map called the Onco-GPS map (28). To assess with which specific RAS cell state, the double-positive cells (PdTm) are associated, we projected them onto the RAS Onco-GPS map. Consistent with the ssGSEA results, we observed that the double-positive cells are associated with a cell state that is associated with the RAS/AP1 pathway (Fig. 6H). Finally, we performed differential gene expression analysis to identify potential therapeutic targets for Kras^G12D^-activated adenocarcinoma by comparing the gene expression of tumor-initiating double-positive cells to type II cells that do not form colonies *in vitro* or initiate tumors upon transplantation in immunodeficient mice (Supplementary Fig. S7). These genes might be responsible for tumor cell phenotype and may be druggable. Our results suggest that these may be the useful potential targets for development of new therapeutics.

## Discussion

We have demonstrated that Kras activation in type II cells leads to a Notch-dependent development of a subset of cells into cells expressing type I and type II markers. These cells are tumor propagating cells *in vitro* and *in vivo*. Their *in vitro* proliferation capability and ability to form tumors *in vivo* are abrogated by Notch inhibition and Sox2 upregulation. RNA-seq analysis of this cellular subset demonstrates transcriptional similarity to published analyses of embryonic lung progenitor cells and identifies potential therapeutic opportunities. We have provided the proposed model of our study in Supplementary Fig. S8.

One tenet of the cancer stem cell hypothesis is that tumors arise from stem cells that acquire genetic mutations or from differentiated cells that acquire stem-like properties as a result of mutations. Other recent publications have also highlighted emergence of less differentiated populations of alveolar cells after Kras activation (18–20). The current study demonstrates that notch activation is necessary for the development of type I markers in alveolar type II cells upon Kras^G12D^ mutation. However, which specific pathway members of Notch are involved in this dedifferentiation process, will be the focus of further studies. Others have identified Notch3 as responsible for maintenance of CD24^+^, ITGB4+ tumor-propagating cells in a Kras model (34) highlighting the possibility that Notch activation is required for this phenotype across models.

In addition, the phenotype of the tumor-propagating cell may differ based upon genotype of the tumor cells (35). It will thus be important to examine whether these dual-positive progenitor cells are present and necessary for tumor formation when additional mutations such as p53 are present. We have previously demonstrated that a transcriptional signature of early (E11.5) versus late (E17.5) embryonic mouse lung distal tip cells predicts prognosis in human patients with lung adenocarcinoma (33). The dual-positive cells described in this study are transcriptionally similar to both of these gene sets. Analyzing the degree to which transcriptional changes identified here are applicable to resected human lung tumors will be a basis of future study.

The ultimate goal of these studies is to develop better therapies for human patients with KRAS-mutant lung adenocarcinoma. We have previously demonstrated that Sox2 antagonizes Notch pathway members when Kras is activated in type II cells. Sox2 also inhibits double-positive cell development and tumor formation in our models. Notch inhibition and RepSox treatment may be developed as therapeutic options for KRAS-mutant lung adenocarcinomas. Our RNA-seq analysis has identified the genes that are differentially expressed between type II and double-positive cells.

The Onco-GPS analysis also identifies potential therapeutics that may be effective against double-positive progenitor-like cells. We have found that these double-positive cells are associated with ERBB3/PI3K and AP1 in OncoGPS. It has been reported that KRAS-mutant human cells associated with ERBB3/PI3K and AP1 in OncoGPS are sensitive to a combination of lapatinib and PD-0325901 (28). We will test lapatinib, PD-0325901 and other predicted combinations in the future. Finally, the ability to grow these dual-positive cells as colonies in *in vitro* will be useful for testing other therapeutics in the future.

## Supplementary Material

Supplementary Figure S1 Time course for double positive cells in Sftpc-creER; KRasG12D; fGFP mice

Supplementary Figure S2 Subcutaneous transplantation of double positive cells (Type I/II+) in Rag1-/- or NOD SCID mice leads to large tumor formation

Supplementary Figure S3 H&E staining of tumors developed from transplant of Type II and double positive cells

Supplementary Figure S4 Tumors from both Type II and double positive cells transplants express both Type I and Type II cells

Supplementary Figure S5 Sox2 overexpressing population in Sftpc-CreER; KrasG12D; LSL-Sox2 mice express proximal markers, CC10 and Krt5

Supplementary Figure S6 RepSox treatment increases Sox2 levels while TGFb remains unchanged in KrasG12D mutant lungs

Supplementary Figure S7 Heat map showing the differentially expressed genes between double positive and Type II cells in KrasG12D induced lungs

Supplementary Figure S8 Proposed model showing transformation of type II cells into lung adenocarcinoma through Rage+/Sftpc+ double positive stage

Supplementary Table S1 Validated alveolar type I and type II epithelial cell markers

Supplementary Table S2 List of the primers used in qPCR analysis
