# Supplementary material for: Activation of KrasG12D in Subset of Alveolar Type II Cells Enhances Cellular Plasticity in Lung Adenocarcinoma
Source: Cancer Res Commun. 2023 Nov 24;3(11):2400–11. doi: 10.1158/2767-9764.CRC-22-0408 (PMC10668634; doi:10.1158/2767-9764.CRC-22-0408)
Supplement: Supplementary Figure S1 — Time course for double positive cells in Sftpc-creER; KRasG12D; fGFP mice [file crc-22-0408-s01.pdf]

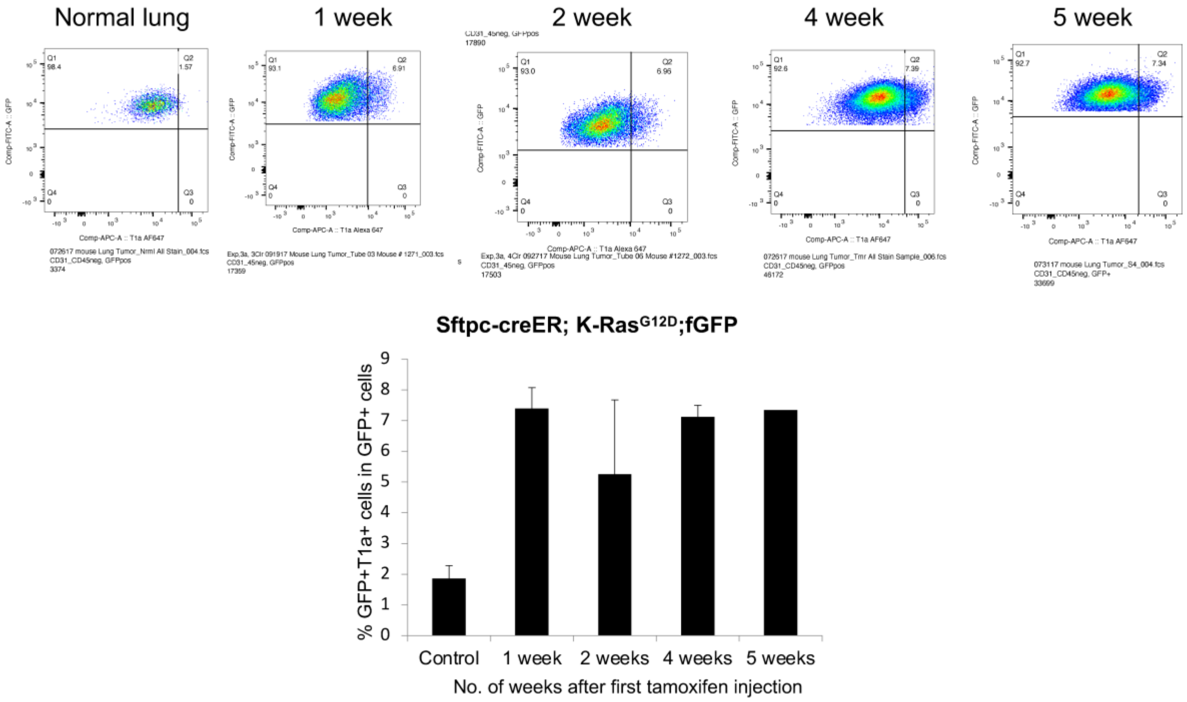

Fig. S1. Time course for double positive cells in Sftpc-creER; KRas^{G12D}; fGFP mice
Sftpc-creER; fGFP (control) and Sftpc-creER; K-Ras^{G12D}; fGFP mice were injected with single dose of tamoxifen. Lungs were harvested at 1 week, 2 weeks, 3 weeks, 4 weeks and 5 weeks post tamoxifen injections and flow cytometry was performed as described in the method sections. Our results shows that activation of Kras causes increase in double positive cells (Type I/II+ (T1a+/GFP+)). The time course study also reveal that increase in double positive population in lung remain same over the time from 1 week post tamoxifen administration to 5 weeks.
