# Supplementary material for: Activation of KrasG12D in Subset of Alveolar Type II Cells Enhances Cellular Plasticity in Lung Adenocarcinoma
Source: Cancer Res Commun. 2023 Nov 24;3(11):2400–11. doi: 10.1158/2767-9764.CRC-22-0408 (PMC10668634; doi:10.1158/2767-9764.CRC-22-0408)
Supplement: Supplementary Figure S2 — Subcutaneous transplantation of double positive cells (Type I/II+) in Rag1-/- or NOD SCID mice leads to large tumor formation [file crc-22-0408-s02.pdf]

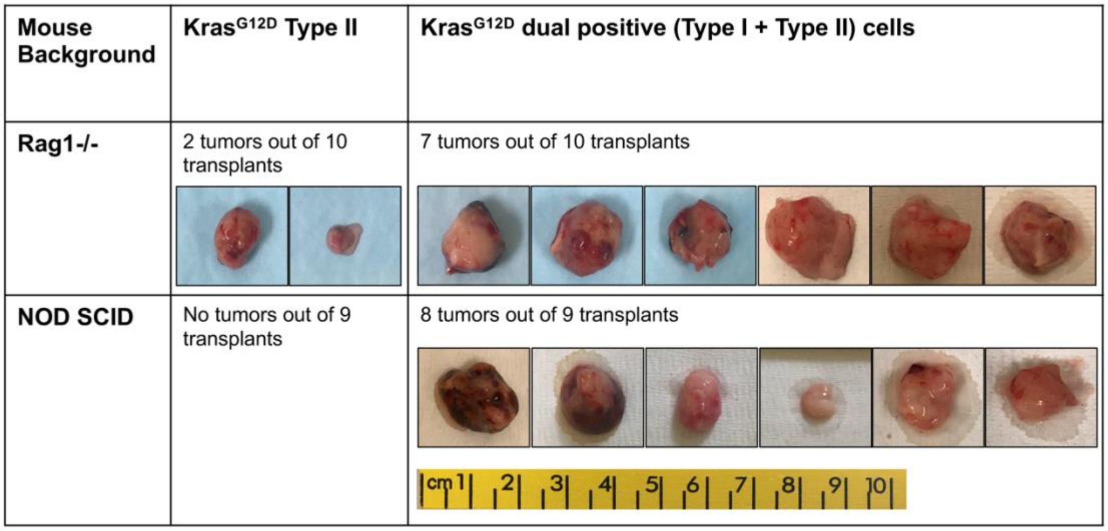

Fig. S2. Subcutaneous transplantation of double positive cells (Type I/II+) in Rag1$^{-/-}$ or NOD SCID mice leads to large tumor formation.

25,000 Type II+ and double positive cells (Type I/II+) were administered subcutaneously over the flank in Rag1$^{-/-}$ and NOD SCID mice. The success rate of tumor development was significantly higher with transplants of ‘double positive cells’ (70% in Rag1$^{-/-}$ and 88% in NOD SCID mice) compared to ‘Type II cells’ (20% in Rag1$^{-/-}$ and 0% in NOD SCID mice). Moreover, tumors obtained from double positive cells were considerably larger than tumors from transplants of Type II cells.
