# Supplementary material for: Activation of KrasG12D in Subset of Alveolar Type II Cells Enhances Cellular Plasticity in Lung Adenocarcinoma
Source: Cancer Res Commun. 2023 Nov 24;3(11):2400–11. doi: 10.1158/2767-9764.CRC-22-0408 (PMC10668634; doi:10.1158/2767-9764.CRC-22-0408)
Supplement: Supplementary Figure S3 — H&E staining of tumors developed from transplant of Type II and double positive cells [file crc-22-0408-s03.pdf]

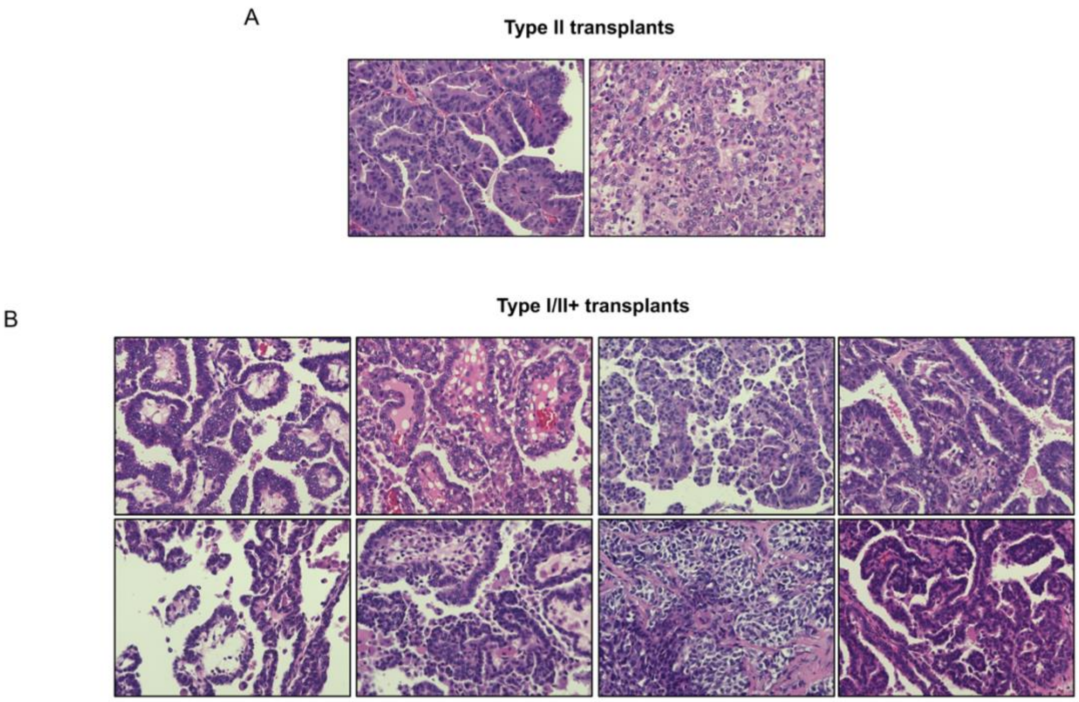

Fig. S3. H&E staining of tumors developed from transplant of Type II and double positive cells.
A) H&E staining was performed on both tumors that were obtained from transplants of Type II cells. At least one of the tumors showed (left panel) adeno-papillary histology. The other tumor didn't have distinct histology.
B) H&E staining of tumors developed from transplants of double positive cells. All tumors showed adeno-papillary histology.
