# Supplementary material for: Activation of KrasG12D in Subset of Alveolar Type II Cells Enhances Cellular Plasticity in Lung Adenocarcinoma
Source: Cancer Res Commun. 2023 Nov 24;3(11):2400–11. doi: 10.1158/2767-9764.CRC-22-0408 (PMC10668634; doi:10.1158/2767-9764.CRC-22-0408)
Supplement: Supplementary Figure S4 — Tumors from both Type II and double positive cells transplants express both Type I and Type II cells [file crc-22-0408-s04.pdf]

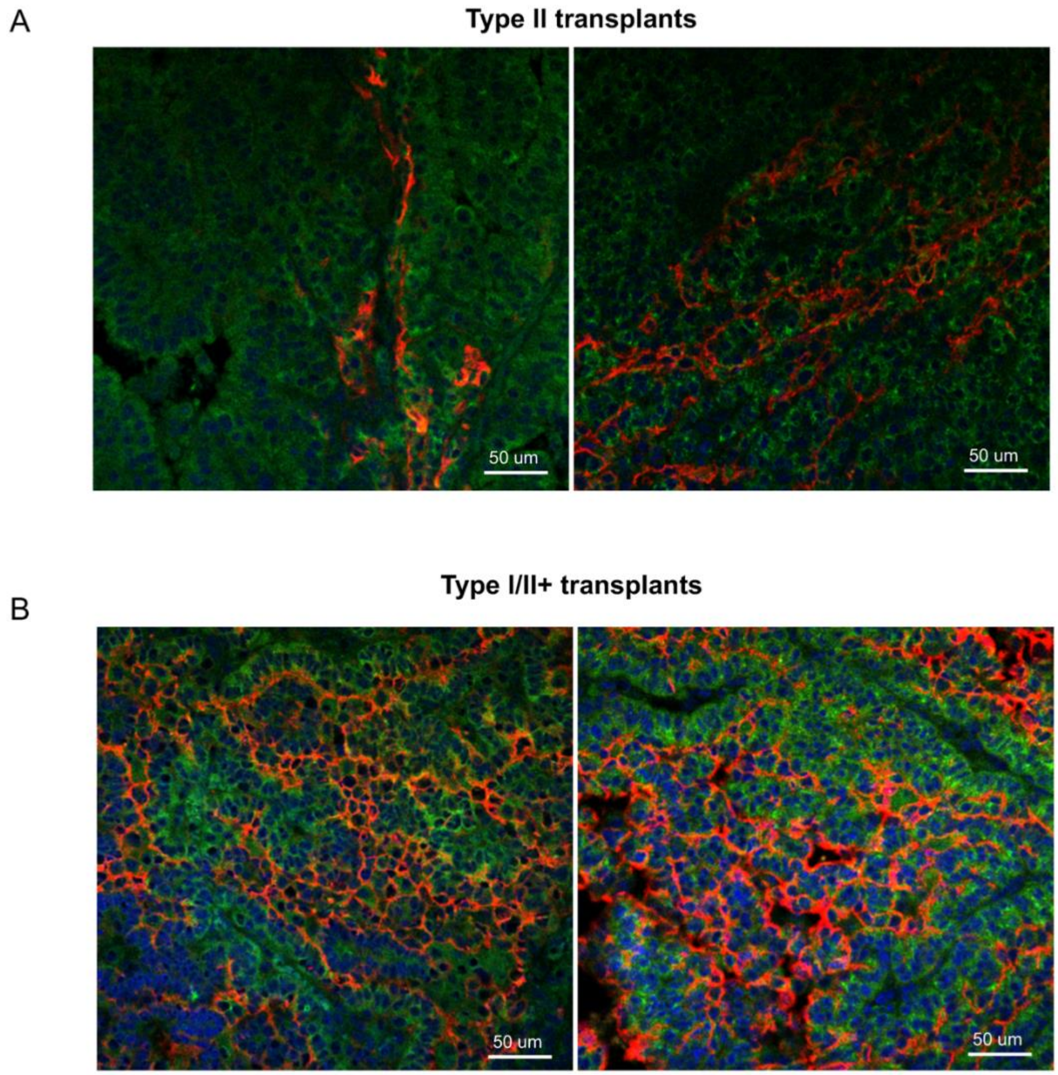

Fig. S4. Tumors from both Type II and double positive cells transplants express both Type I and Type II cells

Immunofluorescence images for tumors from transplants of A) Type II and B) double positive cells. IF analysis indicates that tumors from all transplants express both ‘Type I’ and ‘Type II’ cells, however fewer ‘Type I’ cells are expressed in tumors of transplants from ‘Type II’ cells compared to ‘double positive’ cells. Moreover, ‘Type I’ cells in ‘Type II transplant tumors’ are found in center of tumor while they are widely spread across the tumor in transplants from double positive cells.
