# Supplementary material for: Activation of KrasG12D in Subset of Alveolar Type II Cells Enhances Cellular Plasticity in Lung Adenocarcinoma
Source: Cancer Res Commun. 2023 Nov 24;3(11):2400–11. doi: 10.1158/2767-9764.CRC-22-0408 (PMC10668634; doi:10.1158/2767-9764.CRC-22-0408)
Supplement: Supplementary Figure S5 — Sox2 overexpressing population in Sftpc-CreER; KrasG12D; LSL-Sox2 mice express proximal markers, CC10 and Krt5 [file crc-22-0408-s05.pdf]

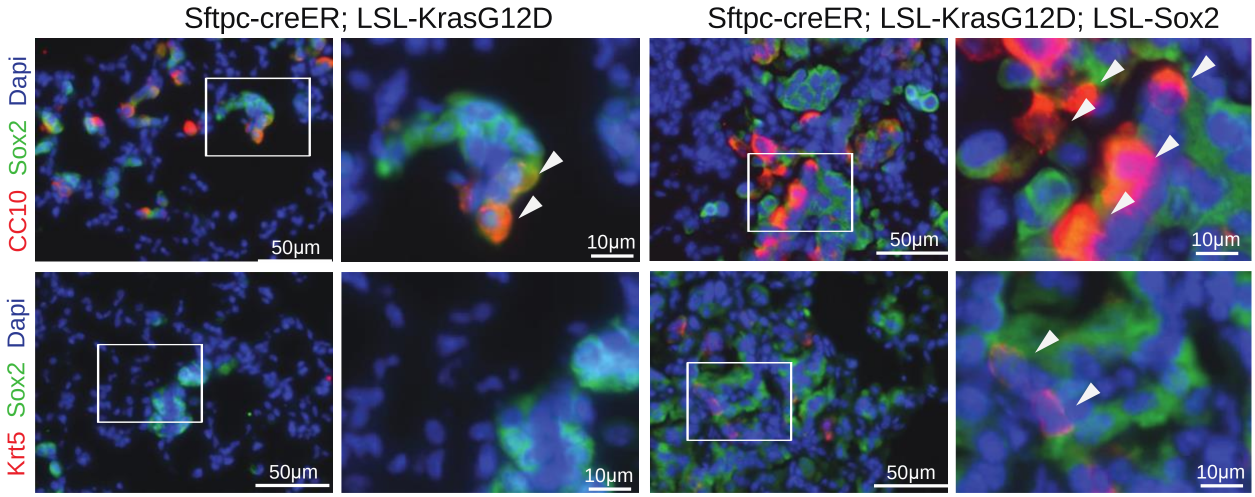

Fig. S5. Sox2 overexpressing population in *Sftpc-CreER; Kras^{G12D}; LSL-Sox2* mice express proximal markers, CC10 and Krt5.

Representative IF images showing CC10 (red) (uppper panels) and Krt5 (red) (lower panels) staining of mouse lung sections in Sftpc-CreER; LSL-KRasG12D and Sftpc-CreER; LSL-KRasG12D; LSL-Sox2 mice. Note the Sox2+ expressing cells (green) express the proximal airway markers CC10 (white arrows, upper panel) and Krt5 (white arrows, lower panels).
