# Supplementary material for: Activation of KrasG12D in Subset of Alveolar Type II Cells Enhances Cellular Plasticity in Lung Adenocarcinoma
Source: Cancer Res Commun. 2023 Nov 24;3(11):2400–11. doi: 10.1158/2767-9764.CRC-22-0408 (PMC10668634; doi:10.1158/2767-9764.CRC-22-0408)
Supplement: Supplementary Figure S6 — RepSox treatment increases Sox2 levels while TGFb remains unchanged in KrasG12D mutant lungs [file crc-22-0408-s06.pdf]

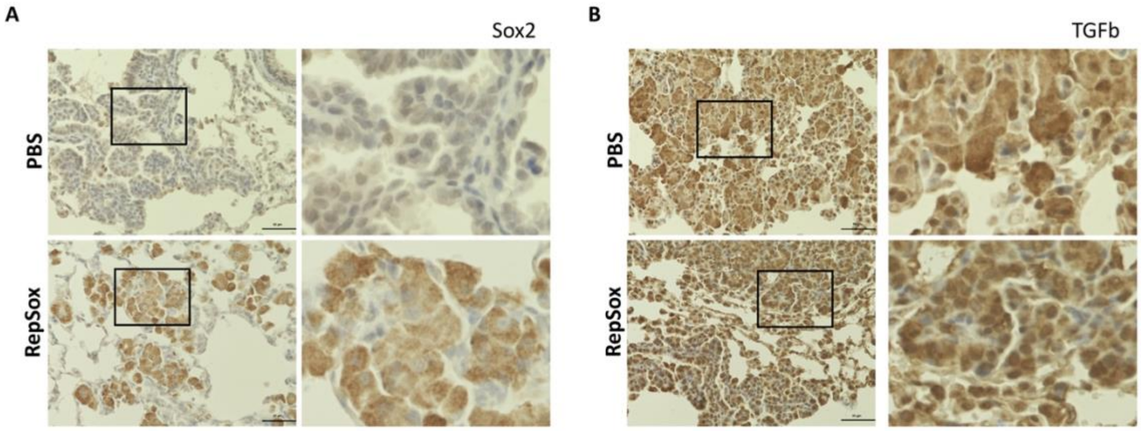

Fig. S6. RepSox treatment increases Sox2 levels while TGFβ remains unchanged in Kras^{G12D} mutant lungs

Immunohistochemistry analysis for A) Sox2 and B) TGFβ upon RepSox treatment in mouse lungs. In order to determine the effect of RepSox on TGFβ and Sox2 levels in our mice model, we have performed immunohistochemistry for Sox2 and TGFβ on PBS vs RepSox treated lung sections from CC10CreER; LSL Kras^{G12D} mice. IHC data shows that there is significant increase in Sox2 expression in RepSox treated lungs. However, no apparent change in the expression of TGFβ was observed between PBS and RepSox treated lung sections from CC10CreER; LSL Kras^{G12D} mice.
