# Supplementary material for: Activation of KrasG12D in Subset of Alveolar Type II Cells Enhances Cellular Plasticity in Lung Adenocarcinoma
Source: Cancer Res Commun. 2023 Nov 24;3(11):2400–11. doi: 10.1158/2767-9764.CRC-22-0408 (PMC10668634; doi:10.1158/2767-9764.CRC-22-0408)
Supplement: Supplementary Figure S7 — Heat map showing the differentially expressed genes between double positive and Type II cells in KrasG12D induced lungs [file crc-22-0408-s07.pdf]

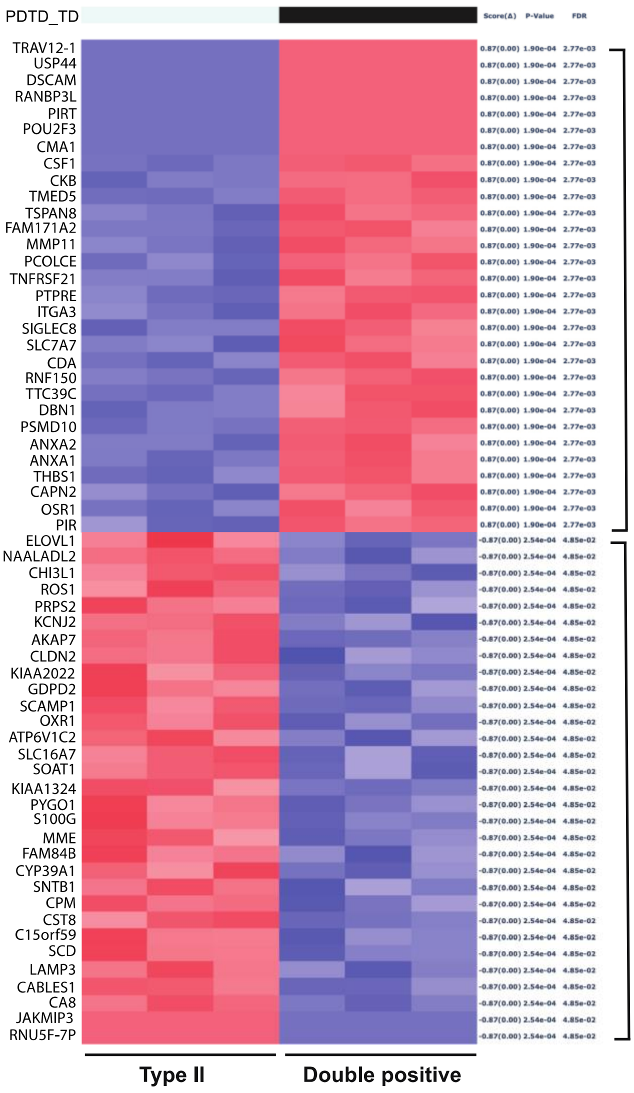

Fig. S7. Heat map showing the differentially expressed genes between double positive and Type II cells in KrasG12D induced lungs.
The upper half represent 30 up-regulated while lower half represent 30 down-regulated genes in double positive cells compared to Type II cells.
