# Supplementary material for: Activation of KrasG12D in Subset of Alveolar Type II Cells Enhances Cellular Plasticity in Lung Adenocarcinoma
Source: Cancer Res Commun. 2023 Nov 24;3(11):2400–11. doi: 10.1158/2767-9764.CRC-22-0408 (PMC10668634; doi:10.1158/2767-9764.CRC-22-0408)
Supplement: Supplementary Figure S8 — Proposed model showing transformation of type II cells into lung adenocarcinoma through Rage+/Sftpc+ double positive stage [file crc-22-0408-s08.pdf]

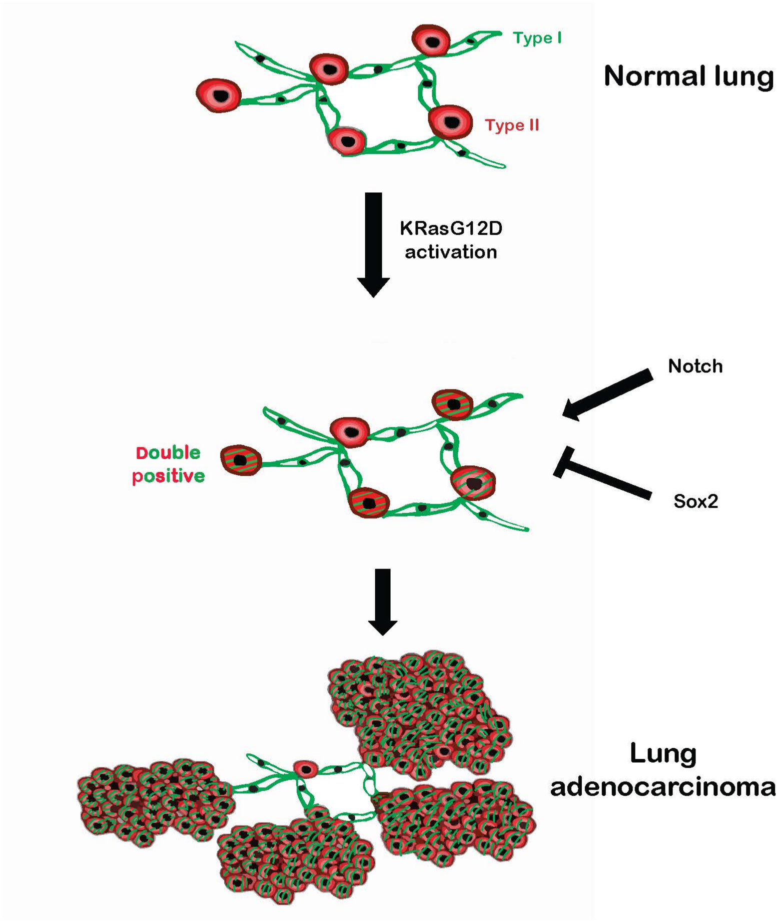


**Fig. S8. Proposed model showing transformation of type II cells into lung adenocarcinoma through Rage+/Sftpc+ double positive stage.**
$Kras^{G12D}$ mutation in type II cells causes development of a subset cells into cells expressing Type I and Type II markers. This process requires notch signaling while Sox2 inhibits it. These dual positive cells are highly plastic and undergo rapid cell-proliferation resulting in hyperplasia, which ultimately leads to lung adenocarcinoma.
