# Supplementary material for: Activation of KrasG12D in Subset of Alveolar Type II Cells Enhances Cellular Plasticity in Lung Adenocarcinoma
Source: Cancer Res Commun. 2023 Nov 24;3(11):2400–11. doi: 10.1158/2767-9764.CRC-22-0408 (PMC10668634; doi:10.1158/2767-9764.CRC-22-0408)
Supplement: Supplementary Table S1 — Validated alveolar type I and type II epithelial cell markers [file crc-22-0408-s09.pdf]

Table S1. Validated alveolar type I and type II epithelial cell markers

AT1		Antigen	Reference
	1	Aqp5	(1)
	2	LEL	(2)
	3	Pdpn (T1α)*	(3)
	4	Rage (Ager)*	(4)
	5	RCA I	(5)
**AT2**		**Antigen**	**Reference**
	1	Abca3	(6)
	2	Ctsh	(7)
	3	Lamp-1, Lamp-2	(8)
	4	Lyz2 (LysM)	(9)
	5	Muc1	(10)
	6	Nkx2.1 (Ttf-1)	(11)
	7	SftpB*	(12)
	8	SftpC*	(13)
	9	SftpD	(14)

* Canonical marker genes

SI References

1. Nielsen S, King LS, Christensen BM, Agre P. Aquaporins in complex tissues. II. Subcellular distribution in respiratory and glandular tissues of rat. Am J Physiol **1997**;273:C1549-61
2. Bankston PW, Porter GA, Milici AJ, Palade GE. Differential and specific labeling of epithelial and vascular endothelial cells of the rat lung by Lycopersicon esculentum and Griffonia simplicifolia I lectins. Eur J Cell Biol **1991**;54:187-95
3. Rishi AK, Joyce-Brady M, Fisher J, Dobbs LG, Floros J, VanderSpek J, *et al.* Cloning, characterization, and development expression of a rat lung alveolar type I cell gene in embryonic endodermal and neural derivatives. Dev Biol **1995**;167:294-306
4. Fehrenbach H, Kasper M, Tschernig T, Shearman MS, Schuh D, Muller M. Receptor for advanced glycation endproducts (RAGE) exhibits highly differential cellular and subcellular localisation in rat and human lung. Cell Mol Biol (Noisy-le-grand) **1998**;44:1147-57
5. Dobbs LG, Williams MC, Brandt AE. Changes in biochemical characteristics and pattern of lectin binding of alveolar type II cells with time in culture. Biochim Biophys Acta **1985**;846:155-66
6. Mulugeta S, Gray JM, Notarfrancesco KL, Gonzales LW, Koval M, Feinstein SI, *et al.* Identification of LBM180, a lamellar body limiting membrane protein of alveolar type II cells, as the ABC transporter protein ABCA3. J Biol Chem **2002**;277:22147-55

7. Ishii Y, Hashizume Y, Kominami E, Uchiyama Y. Changes in immunoreactivity for cathepsin H in rat type II alveolar epithelial cells and its proteolytic activity in bronchoalveolar lavage fluid over 24 hours. Anat Rec **1991**;230:519-23
8. Salaun B, de Saint-Vis B, Pacheco N, Pacheco Y, Riesler A, Isaac S, *et al.* CD208/dendritic cell-lysosomal associated membrane protein is a marker of normal and transformed type II pneumocytes. Am J Pathol **2004**;164:861-71
9. Singh G, Katyal SL, Brown WE, Collins DL, Mason RJ. Pulmonary lysozyme--a secretory protein of type II pneumocytes in the rat. Am Rev Respir Dis **1988**;138:1261-7
10. Jarrard JA, Linnoila RI, Lee H, Steinberg SM, Witschi H, Szabo E. MUC1 is a novel marker for the type II pneumocyte lineage during lung carcinogenesis. Cancer Res **1998**;58:5582-9
11. Ikeda K, Clark JC, Shaw-White JR, Stahlman MT, Boutell CJ, Whitsett JA. Gene structure and expression of human thyroid transcription factor-1 in respiratory epithelial cells. J Biol Chem **1995**;270:8108-14
12. Kalina M, Mason RJ, Shannon JM. Surfactant protein C is expressed in alveolar type II cells but not in Clara cells of rat lung. Am J Respir Cell Mol Biol **1992**;6:594-600
13. Beers MF, Wali A, Eckenhoff MF, Feinstein SI, Fisher JH, Fisher AB. An antibody with specificity for surfactant protein C precursors: identification of pro-SP-C in rat lung. Am J Respir Cell Mol Biol **1992**;7:368-78
14. Crouch E, Rust K, Marienchek W, Parghi D, Chang D, Persson A. Developmental expression of pulmonary surfactant protein D (SP-D). Am J Respir Cell Mol Biol **1991**;5:13-8
