# Supplementary material for: Activation of KrasG12D in Subset of Alveolar Type II Cells Enhances Cellular Plasticity in Lung Adenocarcinoma
Source: Cancer Res Commun. 2023 Nov 24;3(11):2400–11. doi: 10.1158/2767-9764.CRC-22-0408 (PMC10668634; doi:10.1158/2767-9764.CRC-22-0408)
Supplement: Supplementary Table S2 — List of the primers used in qPCR analysis [file crc-22-0408-s10.pdf]

Table S2. List of the primers used in qPCR analysis

Primer	Primer sequence 5 to 3
GAPDH_F<br>GAPDH_R	CTCCCACTCTTCCACCTTCG<br>GCCTCTCTTGCTCAGTGTCC
SftpC_F<br>SftpC_R	ATGGAGAGTCCACCGGATTA<br>ACCACAACCACGATGAGAAG
SftpB_F<br>SftpB_R	GTGGAGCCTCTGATAGAAGAATG<br>ATAGCCTGTTCACTGGTGTTC
SftpD_F<br>SftpD_R	AAGGCTGCTTTCCTGAGTATG<br>CCTGGAGCCCAATTAGAATAGAC
lamp2_F<br>lamp2_R	CTGACTCCTGTCGTTCAGAAAT<br>GGTGGGAGTTTGGTCTTCTT
etv5_F<br>etv5_R	AGTCAGGAGTTCCTGGAGATAG<br>TCAAACAGTGGGTCATGGTATT
aqp5_F<br>aqp5_R	GGCTGCAATCCTCTACTTCTAC<br>TTCTTCCGCTCCTCTCTATGA
rage_F<br>rage_R	GTTGAGCCTGAAGGTGGAATAG<br>AAGGGTGCACCATCCTTTATC
